# Advanced Microfluidic Device Designed for Cyclic Compression of Single Adherent Cells

**DOI:** 10.3389/fbioe.2018.00148

**Published:** 2018-10-16

**Authors:** Kenneth K. Y. Ho, Ying Lin Wang, Jing Wu, Allen P. Liu

**Affiliations:** ^1^Department of Mechanical Engineering, University of Michigan, Ann Arbor, MI, United States; ^2^Department of Mechanical Engineering, University of Hong Kong, Hong Kong, China; ^3^Department of Biomedical Engineering, University of Michigan, Ann Arbor, MI, United States; ^4^Cellular and Molecular Biology Program, University of Michigan, Ann Arbor, MI, United States; ^5^Biophysics Program, University of Michigan, Ann Arbor, MI, United States

**Keywords:** microfluidics, cell mechanics, mechanobiology, compression, single-cell analysis, microcontact printing

## Abstract

Cells in our body experience different types of stress including compression, tension, and shear. It has been shown that some cells experience permanent plastic deformation after a mechanical tensile load was removed. However, it was unclear whether cells are plastically deformed after repetitive compressive loading and unloading. There have been few tools available to exert cyclic compression at the single cell level. To address technical challenges found in a previous microfluidic compression device, we developed a new single-cell microfluidic compression device that combines an elastomeric membrane block geometry to ensure a flat contact surface and microcontact printing to confine cell spreading within cell trapping chambers. The design of the block geometry inside the compression chamber was optimized by using computational simulations. Additionally, we have implemented step-wise pneumatically controlled cell trapping to allow more compression chambers to be incorporated while minimizing mechanical perturbation on trapped cells. Using breast epithelial MCF10A cells stably expressing a fluorescent actin marker, we successfully demonstrated the new device design by separately trapping single cells in different chambers, confining cell spreading on microcontact printed islands, and applying cyclic planar compression onto single cells. We found that there is no permanent deformation after a 0.5 Hz cyclic compressive load for 6 min was removed. Overall, the development of the single-cell compression microfluidic device opens up new opportunities in mechanobiology and cell mechanics studies.

## Introduction

Cells and tissues in our body experience various kinds of chemical and mechanical signals in physiological and pathological conditions. Due to the complex environment and multiple interactions with neighboring cells, cells experience different combinations of compressive, tensile and shear stresses in different directions. Most living cells exhibit viscoelastic deformation under mechanical stress (Fabry et al., 2001). When the mechanical load is removed, the cell usually recovers partially to its original undeformed shape. The incomplete shape recovery is mainly due to the rupture of bonds within the cytoskeleton when the cells experience tensile stress, leading to the observed plasticity (Bausch et al., 2001; Bonakdar et al., 2016). Repetitive tensile loading and unloading was found to result in an increase in residual deformation, which was suggested to be an adaptive process for cells to protect themselves against mechanical damage (Bonakdar et al., 2016). However, little is known how cells respond to repetitive compressive loading and unloading.

The study of cellular responses to compression, tension and shear has a long history, particularly in musculoskeletal (Grodzinsky et al., 2000) and vascular (Shyy and Chien, 2002; Gupta and Grande-Allen, 2006) tissues. In many cases, bioengineering tools have played a central role in deciphering mechanotransduction pathways (Polacheck et al., 2013; Liu et al., 2017). However, there is a growing interest in studying the response of cells from compressive stress in other physiological environments, for example during development (Mammoto and Ingber, 2010) and in cancer (Jain et al., 2014; Ricca et al., 2018). Mechanical forces, such as compression generated by living cells are crucial for the control of embryonic development. Solid stress is developed in tumor microenvironment because uncontrolled proliferation of cancer cells leads to an increase in compressive stress. Dense extracellular matrix and endothelial barriers also present physiological scenarios where cells experience significant compression (Reymond et al., 2013). Several recent studies have utilized microfabricated channels with narrow constriction that lead to compression as cells migrate in these microchannels (Denais et al., 2016; Raab et al., 2016; Heureaux et al., 2018). Different structures inside the cells, such as actin cortex beneath the plasma membrane, vertical actin fibers connecting the apical and basal surfaces of the cells and the mechanical stiffness of the nucleus, are responsible for withstanding planar compressive deformation. Thus, cells with different stiffnesses, particularly between healthy and diseased cells, may respond differently to compression. Despite a general understanding, how cells withstand and respond to planar compression is not as well-understood compared to cellular responses to tension or shear. The development of a microengineering device that applies uniform and well-controlled compression will aid the investigation of how cells recover after compression.

Different experimental techniques were developed to apply compression on cells (Van Vliet et al., 2003). Modified atomic force microscopy (AFM) probes was developed to apply compressive forces to single cells (Lulevich et al., 2006, 2010; Rosenbluth et al., 2006; Weafer et al., 2013). While AFM is a powerful approach to apply compressive forces and measure deformation of cells, this sophisticated method has a low throughput (e.g., one cell at a time) and requires expensive equipment and technical expertise. Microfluidics holds great promise as a next generation tool for mechanically perturbing single cells (Liu, 2016). With the integration of microsized and fast-operating valves in the microfluidic system, several microfluidic platforms have been developed for studying biological responses of cells under a compressive stress (Kim et al., 2007; Hosmane et al., 2011; Si et al., 2015). These microengineering devices allow the application of compression to cells.

Our lab previously developed a microfluidic aspiration and compression device and demonstrated compression of double emulsion droplets (Ho et al., 2016). However, there were two critical challenges that prevented the use of the same device for single-cell compression. First, the concave deflection profile of the polydimethylsiloxane (PDMS) membrane does not provide a controlled contact area between the membrane and the cells (Figure S1A), thus affecting the forces applied to each cell. Second, despite the high cell trapping efficiency, cells randomly spread inside the microfluidic device and were rarely directly underneath the deflection membrane for compression (Figure S1B).

To overcome the first challenge of the concave PDMS membrane deflection profile, we spatially varied bending rigidity across the membrane by increasing the thickness of the membrane in the middle. This was accomplished by including a block of PDMS in the middle of the membrane. In this case, the membrane in the middle has a higher resistance to deform comparing to the membrane at the side. This creates a flat deflection profile in the middle of the membrane, while having a concave deflection profile on the side. To control where cells spread within the compression chambers, we implemented microcontact printing of fibronectin islands that are positioned directly underneath the deflection membrane. In this work, we describe our effort in developing a microfluidic device for single-cell static and cyclic compression. We will first describe the design process and experimental validation of the block in the PDMS membrane. Then, we explain our newly designed two-step, pneumatically controlled cell trapping to further facilitate trapping. With the fabrication process flow, we demonstrated the alignment of the microcontact printed fibronectin island to control the cell spreading location. Finally, we showed the device capability to apply cyclic compression on cells and found that there is no permanent change in the height of the breast epithelial MCF-10A cells after cyclic compression.

## Materials and methods

### Device overview and design

**Figure 1:**
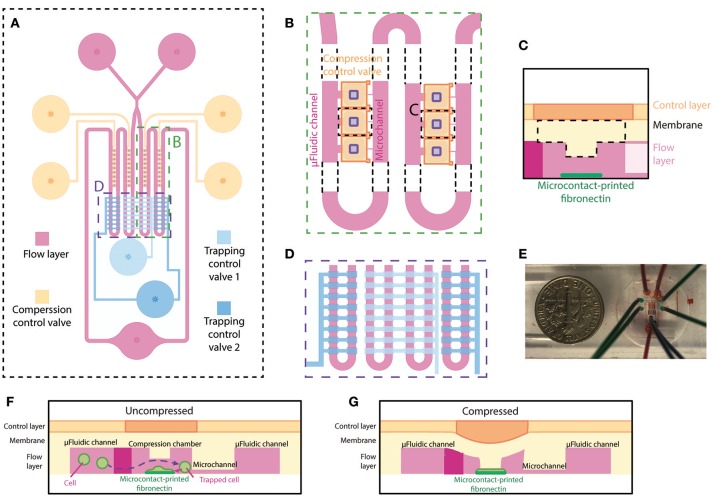
Overview and design of the microfluidic device for single-cell compression. **(A)** The top view of overall design of the device. The flow layer is labeled in magenta, and the control layer consists of trapping control valves (blue) and compression control valve (orange). **(B)** The zoomed-in top view of the compression chamber (marked in A) and the meandering microfluidic channel in the device. The orange compression control valve is on top of the compression chamber in the flow layer. The purple rectangles are the designed block attached to the deflection membrane for compression. **(C)** Side view schematic of the compression chamber. A rectangular block and fibronectin island are unique features of this device. **(D)** The zoomed-in top view of the main meandering microfluidic channel (marked in A) and trapping control valve (blue). The inside two columns and outside two columns are controlled separately by two different trapping control valves. **(E)** A picture of the device. **(F,G)** Side view schematics of the device when the compression control valve is uncompressed **(F)** or compressed **(G)** on a cell spread on the fibronectin island.

The microfluidic device, made out of PDMS, is specifically designed for single-cell compression. The microfluidic device is designed to trap single cells in different chambers, confine cell spreading on microcontact printed islands, and apply planar compression onto the cells. The device consists of two layers, the flow layer (magenta) and the control layer (blue and orange) (Figure 1A), similar to the previously designed microfluidic device in our group (Ho et al., 2016). The flow layer has a comparable design, where fluid and cells flow from two inlets through the microfluidic channel to one outlet. An extra inlet was added to reduce the possibility of air bubble injection during addition of cell suspension and cell culture medium. In our current implementation, the device has four columns each containing 9 compression chambers. The compression chamber provides a shear-free space for cells to spread and be compressed. Each compression chamber is connected to the opposing end of the main microfluidic channel through a small microchannel (Figure 1B). The dimensions of microfluidic channel are designed so that the flow resistance of the main microfluidic channel is 20 times smaller than that of the small microchannel. This allows the majority of fluid to flow into the main microfluidic channel and that less pressure is applied to the cells when they are trapped inside the compression chamber. A thin PDMS membrane separates the microfluidic channel (flow layer) and the control layer. The center of the membrane inside the compression chamber is designed to be thicker by adding a rectangular block underneath the membrane for applying planar compression (Figure 1C). Fibronectin is microcontact-printed underneath the block so that the cell spreads in the middle of the compression chamber (Figure 1C). Two pneumatically controlled valve sets, trapping control valve (light and dark blue) and compression control valve (orange), are located above the main microfluidic channel and above the compression chambers, respectively. These two sets of microfluidic control valves contained two and four independent inlets, respectively. Each controlled valve set is independently controlled using a pressure regulation setup (with an electro-regulator, Proportion-Air, QBX and a pressure regulator, Norgren R07-200-RGEA) to direct flow to the compression chambers or to compress cells. Two trapping control valves feature rectangular patterns across the main microfluidic channels for controlling cell capture (Figure 1D). The main microfluidic channels with trapping control valves are shown in Figure 1E in different color dyes. Compression control valve features rectangular patterns directly above each compression chamber for compressing cell (Figures 1F,G).

### Imaging

A spinning disk confocal microscope (Olympus IX73 with Yokogawa CSU-X1) or an epi-fluorescence microscope (Nikon, Ti Eclipse) were used for brightfield and fluorescence imaging. The spinning disk confocal microscope has an Andor IXON DU897 EMCCD camera and the epifluorescence microscope has a Hamamatsu Flash 4 cMOS camera.

For measuring cell size and height, z-stack fluorescence confocal images were acquired using the spinning disk confocal microscope and the images were reconstructed in ImageJ to generate side view images of the cells.

### Preparation of cell lines

Non-tumorigenic breast epithelial cell MCF-10A cells were cultured in growth media DMEM premixed 1:1 with Ham's F12 nutrient mixture with 5% horse serum, 1% pen-strep, 2.5 μg/ml amphotericin B (fungizone), 5 μg/ml gentamycin, 10 μg/ml insulin, 0.5 μg/ml hydrocortisone, 0.02 μg/ml epidermal growth factor, and 0.1 μg/ml cholera toxin at 5% CO_2_ and 37°C until about 70% confluency. 5 μg/ml Hoechst dye in PBS was used to label the nucleus. Stable cell lines expressing eGFP and Lifeact-RFP were generated *via* lentiviral transduction for labeling the cell volume and filamentous actin, respectively. Cells were resuspended at 10^6^ cells/ml in the growth media to minimize cell clumping and possible pressure fluctuation during the experiment due to clumped cells blocking up small channels.

### Membrane deflection simulation

Membrane deflection in the compression chamber of the microfluidic device was simulated using COMSOL 4.4 (COMSOL Multiphysics). The simplified three-dimensional model of the membrane and block was constructed in COMSOL and was simulated using the solid mechanics module. PDMS was modeled as a linear elastic material with elastic modulus of 0.3 MPa, a Poisson's ratio of 0.49 and a density of 970 kg/m^3^. A uniform pressure of 10 psi was applied as boundary load on top of the membrane, while the four sides of the membrane were fixed.

The three-dimensional model of the complete device model was constructed in Solidworks. The deflection of the membrane and the block was simulated using COMSOL 4.4 with the same simulation module, material properties, and pressure applied as in the membrane deflection simulation.

### Device fabrication–PDMS casting

The microfluidic device was fabricated using multilayer soft lithography technique (Xia and Whitesides, 1998). The SU-8 patterning of the four silicon molds were described in the Supplementary Material. The microfluidic device is composed of a PDMS control layer, a PDMS flow layer and a fibronectin printed, PDMS-coated glass coverslip, which were sequentially aligned and bonded permanently together. Schematic of the fabrication process flow of the microfluidic device is illustrated in Figure S2.

Before PDMS casting or spin-coating onto the silicon molds, all four wafers were first oxygen plasma-treated and then silanized with trichloro(1H,1H,2H,2H-perfluorooctyl)silane (Sigma-Aldrich) in a desiccator for 2 h or overnight. The silicon mold for the control layer was casted with PDMS (Sylgard-184) with a mixing ratio of 7:1 (base:curing agent), while both the silicon mold for the bottom alignment layer and the microcontact printing layer were casted with PDMS with a mixing ratio of 10:1. After degassing in a desiccator, the control layer, bottom alignment layer and microcontact printing layer PDMS substrate were then cured at 60°C overnight before demolding from the wafer. The control layer PDMS substrate was then diced and holes were punched with 1 mm diameter at the inlets of the microfluidic control valves, while the bottom alignment layer and microcontact printing layer PDMS substrates were also diced. The flow channel membrane was generated by spin-coating PDMS with a mixing ratio of 20:1 (base:curing agent) on the flow layer silicon mold at rotational speeds 1,200 rpm for 60 s. After this, the PDMS flow layer membrane was cured at 60°C for 2 h. The membrane thickness was measured using a stylus profilometer (Dektak 6M). Both the diced PDMS control substrate and the PDMS flow layer membrane on the silicon mold were placed in an oxygen plasma etcher (Femto, Covance) to render the PDMS surfaces hydrophilic for the preparation of bonding procedure described as follows. The flow layer silicon mold containing the PDMS membrane was mounted on a customized alignment platform on an optical microscope. The diced PDMS control layer substrate was then carefully aligned and bonded with the PDMS flow layer membrane. Permanent bonding between the control layer substrate and PDMS flow layer membrane was achieved by heating in the oven at 60°C overnight with the aid of gentle pressing between the two substrates.

The day after, the bonded control layer substrate with the flow layer membrane was then cut out and peeled off from the flow layer silicon wafer. Inlet and outlet holes (1 mm diameter) for the main microfluidic flow channel were punched through the layer PDMS control/flow substrate. The bottom alignment substrate which had the similar channel of flow layer was used to align the fibronectin with flow layer. First, PDMS microcontact printed substrate (see *Microcontact Printing* section) was aligned with the bottom alignment substrate to print the fibronectin on a PDMS-coated glass coverslip. Then the PDMS microcontact printed substrate was removed. Immediately after, the PDMS control/flow substrate was placed in an oxygen plasma etcher to render the PDMS surface hydrophilic before aligning with the bottom alignment substrate and permanently bonding to the fibronectin-printed glass coverslip. The device was kept in 4°C until use.

### Microcontact printing

To confine single MCF-10A cells, we used circular micropatterns with 16 μm diameter (area of 800 μm^2^). 40 μg/ml fibronectin in PBS was incubated on the PDMS stamp for 1–2 h to coat fibronectin on its surface. Then, the PDMS stamp was dried with an air gun. A PDMS-coated coverslip was oxidized by UV-ozone. The bottom alignment substrate would be placed under a PDMS-coated coverslip and aligned with a fibronectin-coated PDMS stamp. The fibronectin is printed onto the PDMS-coated coverslip when the pattern comes into conformal contact with it. We used a mixture of fibrinogen conjugated with Alexa Fluor 647 (Thermo Fisher Scientific, F35200) and fibronectin in a ratio of 1:10 and checked the positions of fibronectin island by fluorescence imaging under a spinning disk confocal microscope. The fibronectin-printed surface was washed with 0.1% (w/v) Pluronic F127 solution for 1 h to passivate the remaining surface and then washed with PBS for 1 h.

### Imaging membrane deflection and 3D image reconstruction

A dilute solution of rhodamine succinimidyl dye (Fisher scientific, 50-851-056) was perfused into the device in order to characterize membrane deflection as a function of applied pressure. The dye solution was imaged using spinning disk confocal microscopy at 20× magnification. The control layer inlets (compression control valve) of the microfluidic device was connected to the pressure regulation setup. The membranes above the chambers were deflected by changing the air pressure in the compression control valve between 0 and 30 psi. A z-series of fluorescence images, excited at 561 nm, was captured at a step size of 500 nm and was reconstructed in ImageJ to generate 3D and side view images.

### Visualization of flow streamlines

1 μm Y (yellow)-G (green) fluorescent beads (Invitrogen; 1:1,000 dilution in DI water) were introduced into the device at a flow rate of 1 μl/min and were imaged at 500 ms exposure to observe the flow streamlines. The PDMS microfluidic device was perfused with DI water first to eliminate any trapped air bubbles before use. Fluorescence images were captured using an epi-fluorescence microscope.

### Two-step, pneumatically controlled cell trapping

The PDMS microfluidic device was perfused with warm medium before MCF-10A cells at a concentration of 10^6^ cells/ml were introduced into device at 0.5–1 μl/min for trapping single cells. To trap cells in the trapping chambers, trapping control valve 1 was pressurized at 20–30 psi to block the first column of the main microfluidic channel, which increased the flow resistance. This led to a change in the flow profile to direct the flow to the compression chambers of the first column, thereby trapping cells. After cells were trapped in the first column, trapping control valve 1 was set back to 0 psi immediately, while trapping control valve 2 was pressurized at 20–30 psi to block the second column of the main microfluidic channel. After cells were trapped in the second column, trapping control valve 2 was set back to 0 psi immediately.

### Cell seeding and compression

After the cells were trapped, heights of the inlet and outlet tubings were adjusted to direct the cells to the middle of each compression chamber by hydrostatic pressure-induced flow. Then all the inlet and outlet tubings were clamped to stop the flow. The device was placed in an incubator for 4 h to allow cell attachment and spreading on the fibronectin-patterned surface. The pressure regulation setup was controlled by a computer program to modulate the pressure. Pressure from 0 to 15 psi was applied to the compression control valve that deflected the membrane for cell compression. Each pressure was maintained for 3 min and z-series of fluorescence images were acquired at excitations of 488, 561, and 405 nm with 500 ms exposure time, at a step size of 500 nm.

### Imaging cyclic membrane deflection

The cyclic membrane deflection was imaged in a similar way as described above. The fluorescence images of the rhodamine succinimidyl dye and the brightfield images of the block in the membrane were captured using spinning disk confocal microscopy at 20× magnification. The microscope was set to image at a time interval of 0.3 s. The compression control valve inlets were connected to the pressure regulation setup with an alternation of pressure between 0 and 10 psi at 0.25 and 0.5 Hz. The timelapse images were reconstructed into a real time video using ImageJ. The fluorescence intensity at the middle of the compression chamber was measured using ImageJ.

### Cyclic compression on live cells

Cyclic compression was applied on cells with the pressure of the compression control valve alternating between 10 and 15 psi at 0.5 Hz for 6 min. The cell height before compression and 6 min after cyclic compression were measured using the reconstructed side view images from the z-stack images.

## Results and discussion

### Optimization of compression chamber design

**Figure 2:**
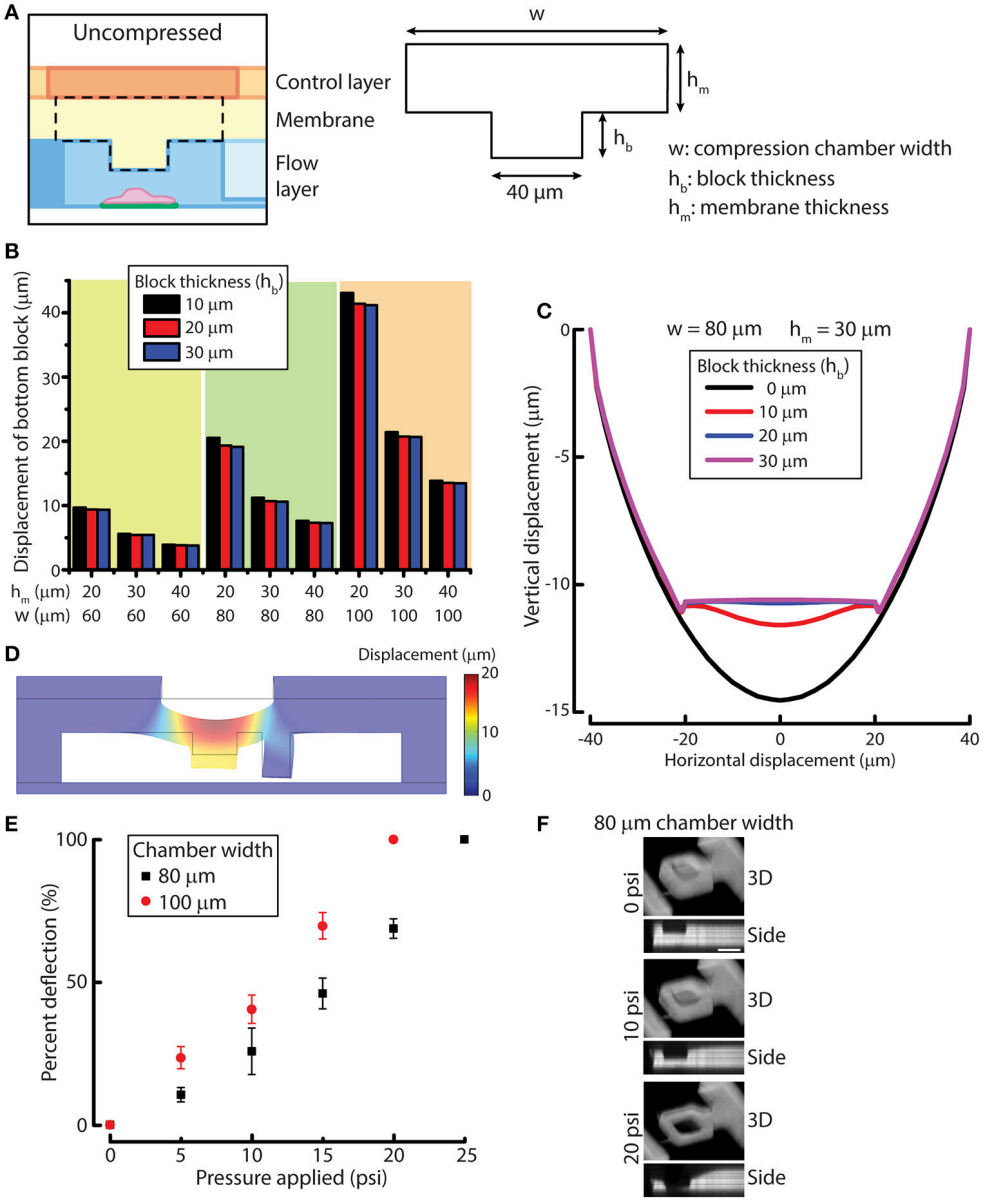
Simulation and experimental results for designing and verifying the PDMS membrane block design of the device for cell compression. **(A)** A simplified model of membrane and block for simulation. Three geometric parameters were defined: compression chamber width (*w*), block thickness (*h*_*b*_) and membrane thickness (*h*_*m*_). **(B)** Displacement of the simplified membrane and block model at different values of geometric parameters. **(C)** Vertical displacement of the membrane and block at different horizontal positions across the compression chamber with different block thicknesses. **(D)** Displacement of the simulated result for the complete device model. **(E)** Membrane deflection of different compression chamber widths as a function of applied pressure. *n* = 3 for each chamber width. Error bar denotes the standard error of mean. **(F)** Reconstructed 3D and side view images of 80 μm chamber width at different compression control valve pressures. Scale bar = 40 μm.

To design the compression chamber for applying uniform compression on cells (e.g., MCF-10A cells), we sought to optimize four different geometric parameters of the compression chamber by using simulation. The membrane and the block in the compression chamber can be characterized by block width, compression chamber width (*w*), block thickness (*h*_*b*_) and membrane thickness (*h*_*m*_) (Figure 2A). In order to design a device that is suited for compressing MCF-10A cells, we first measured MCF-10A cell size and height in suspension and attached on a substrate (Figures S3A,B). The size and height of free or attached MCF-10A cells provide a guidance for determining the geometric parameters of the device. Attached MCF-10A cells spread over 20–40 μm lengthwise. Therefore, the block width was set at 40 μm to ensure that the cells are spread underneath the block even there is some misalignment of the microcontact printed surface during device fabrication. MCF-10A cells in suspension have an average height of 18.8 μm, while attached MCF-10A cells have an average height of 14.1 μm (Figure S3C). The separation between the block and the bottom cell attachment surface was set to be around 20–25 μm to accommodate for the size of MCF-10A in suspension. While the height of attached MCF-10A cells is around 14 μm, we desired the optimal membrane deflection to be around 10–15 μm when a pressure of 10 psi is applied, yielding a cell compression of 4 μm for an average cell.

To optimize the other three design parameters for compression, we performed solid mechanics simulation of a simplified membrane and block model using COMSOL (see Methods for detail). PDMS membrane was modeled as a linearly elastic material. It was shown previously that PDMS with base and curing agent ratio of 20:1 is linearly elastic even under large deformation (Li et al., 2016). The block displacements were determined for 27 conditions where we permutated the values of the three variables (Figure 2B). Based on the simulation results, the compression chamber width and membrane thickness were set as 80 and 30 μm, respectively to achieve a membrane deflection of around 10–15 μm. For the block thickness, we plotted the displacement of the membrane and block with respect to the horizontal position across the compression chamber (Figure 2C). We can readily see that the original concave deflection profile (black line, 0 μm) became flat when the block thickness was increased to 20 μm (blue line) or higher. Therefore, the block thickness was set at 20 μm.

After the block width, compression chamber width, block thickness and membrane thickness were set at 40, 80, 20, and 30 μm, respectively, we performed simulation of the complete device model to evaluate if the deformation of membrane behaves as we expect. At a pressure of 10 psi, the membrane and block deflected between 12 and 13 μm across the block as designed (Figure 2D), suggesting that the simulated results of the simplified membrane and block model can be used to provide design guidelines for fabricating the microfluidic device.

We next examined membrane deflections as a function of different applied pressures, using the optimized parameters determined from our simulation studies. As a comparison, we also fabricated a device with a compression chamber width of 100 μm (instead of 80 μm) while keep all the other parameters the same. The microfluidic channel volume was labeled with rhodamine succinimidyl dye since the PDMS membrane cannot directly be labeled easily. When compression control valve was pressurized, the membrane deflected and displaced the fluid in the compression chamber so that we could indirectly visualize membrane deflection. The 3D and side view images of the compressed compression chamber showed the increase in membrane deflection with increasing compression chamber widths (Figure 2E), where the deflection was greater in 100 μm chamber than in 80 μm chamber, as expected. The thickness of the PDMS membrane spun-coated on the flow layer at 1,200 rpm was 36.1 ± 3.0 μm, which was slightly thicker than the designed membrane thickness of 30 μm. Therefore, membrane deflection in the 80 μm chamber, 6.2 μm at 10 psi, was smaller than designed. However, membrane deflection at 15 psi reached 11.0 μm, fulfilling the design requirement. More importantly, when the membrane deflected, the bottom of the block remained flat while the side membrane is concave (Figure 2F), demonstrating the block design was effective at providing a uniform compression.

### Two-step, pneumatically controlled cell trapping

Due to the increased number of trapping chambers compared with a previous design (Lee et al., 2016), the pressure difference between different chambers of each column will become larger and might affect the trapping efficiency or result large pressure applied to trapped cells. To increase the chances of a trapped cell landing on the fibronectin-printed island, we included more compression chambers in the present design by including two more columns of trapping chambers. To accommodate the new design, the trapping control valve is separated into two individually controlled sets: one controlling the first column (trapping control valve 1) and the other one controlling the second column (trapping control valve 2) of the main microfluidic channel (Figure 1D).

**Figure 3:**
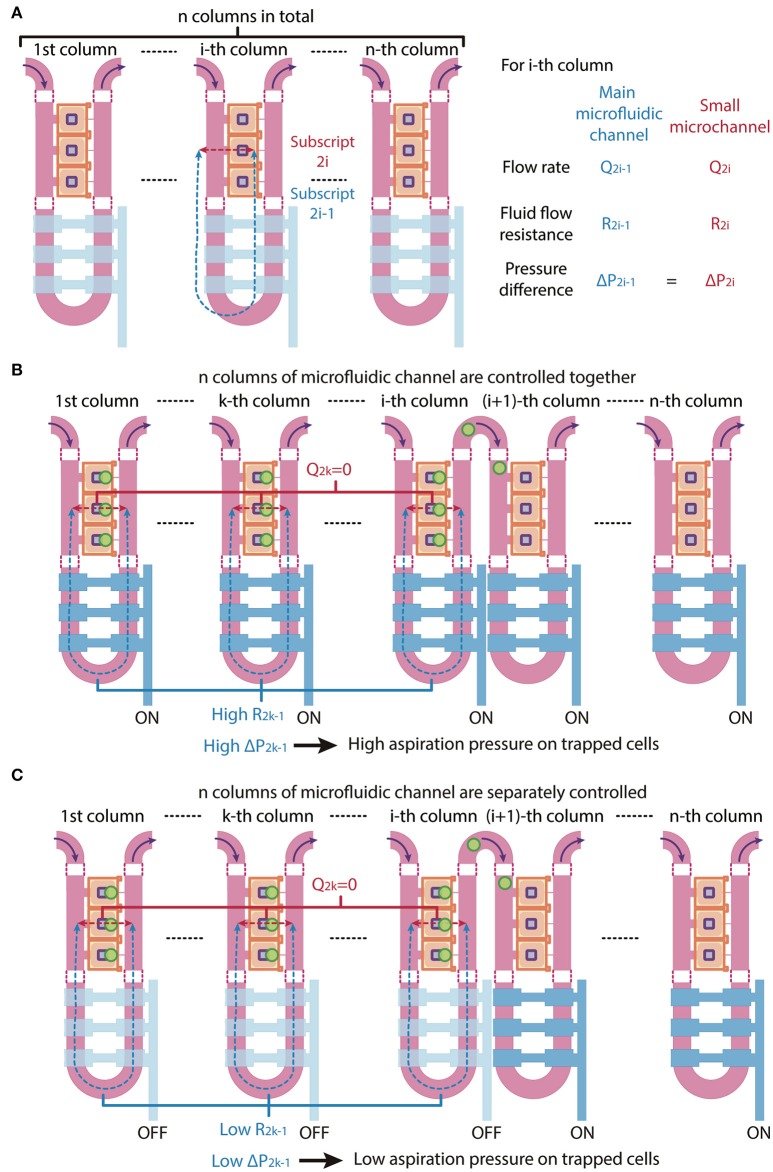
Schematic of multi-column, separately-controlled cell trapping. **(A)** A schematic of a microfluidic channel with *n* individually controlled columns. Each column consists of two channels, so subscripts 2*i* and 2*i*-1 (with *i* = 1,2,3,…, *n*) address individual channels. Pressures (*P*), flow rates (*Q*), and resistances (*R*) of the main microfluidic channel and the small microchannel were denoted in blue and red and are shown in the figure as blue and red dotted lines, respectively. **(B,C)** A schematic of the same microfluidic channel when the *n* columns of microfluidic channel are **(B)** controlled altogether (i.e., all values are *ON*) or **(C)** separately controlled (i.e., valves 1 to *i* are *OFF* and valves *i* + 1 to *n* are *ON*). The consequential changes in fluid flow resistance and pressure difference across cells are shown.

Since the microfluidic device is symmetric we consider one side in our analysis for simplicity. The trapping control valve 1 controls the first column of the main microfluidic channel, while the trapping control valve 2 controls the second column of the main microfluidic channel. In theory, the main microfluidic channel can meander into *n* columns and the trapping control valve can be separated into *n* individually controlled sets, with each set controlling each column of the main microfluidic channel, where *n* is larger than 1. In the following, we will describe volume flow rate as *Q*, fluid flow resistance as *R* and pressure difference as Δ*P*. The subscripts under *Q, R*, and Δ*P* denote the path in which *2i – 1* refer to the main microfluidic channel and *2i* refer to the small microchannel of the *i*-th column, where *i* = *1, 2, 3, …, n* (Figure 3A).

When all *n* control sets are not actuated, the main microfluidic channel will have a 20-times smaller resistance than the small microchannel connecting the compression chamber and the main microfluidic channel on the other side. By the least flow resistance theory, the volume flow rate through the main microfluidic channel *Q*_2*i*−1_ will be higher than the volume flow rate through the small microchannels *Q*_2*i*_ in the *i*-th column, respectively (Figure 3A). Therefore, the volume flow rate ratios *Q*_2*i*−1_/*Q*_2*i*_ should exceed 1.

If all *n* columns of main microfluidic channel are controlled by a single valve, the increase in fluid flow resistance of the main microfluidic channel will lead to a reduction of the volume flow rate ratios *Q*_2*i*−1_/*Q*_2*i*_ of each column at the same time. Since cell loading occurs sequentially, while waiting for cells to be trapped in the *(i* + *1)*-th column of chambers, high fluid flow resistance in the *k*-th column (*k* is a number less than *i*) of the main microfluidic channel *R*_2*k*−1_ will result in a high pressure difference across two sides of the small microchannels in the *k*-th column Δ*P*_2*k*−1_, hence a high aspiration pressure on trapped cells in the *k*-th column, where *k* = *1, 2, …, i* and *i* = *1, 2, …, n – 1* (Figure 3B). This might result a difference in mechanical perturbation to difference cells between compressing them.

We next consider a scenario where the *n* control sets are separately controlled. When only the *i*-th control set is actuated to block the *i*-th column of the main microfluidic channel, only volume flow rate ratio *Q*_2*i*−1_/*Q*_2*i*_ will reduce and only cells in the *i*-th column of chambers will be trapped. After one cell is trapped in each chamber of the *i*-th column, the *i*-th control set is turned to OFF and only then is the *(i* + *1)*-th control set turned to ON. In this case, only volume flow rate ratio *Q*_2*i*+1_*/Q*_2*i*+2_ will reduce and cells will be trapped in the (*i* + *1)*-th column of chambers. Thus, Δ*P*_2*k*−1_ can be kept as minimum, since *R*_2*k*−1_ and *Q*_2*k*−1_ are both small, where *k* = 1, 2, …, *i* and *i* = *1, 2, …, n – 1* (Figure 3C). With the *n* control sets are separately controlled, trapped cells experience less mechanical perturbation while other cells are being trapped.

**Figure 4:**
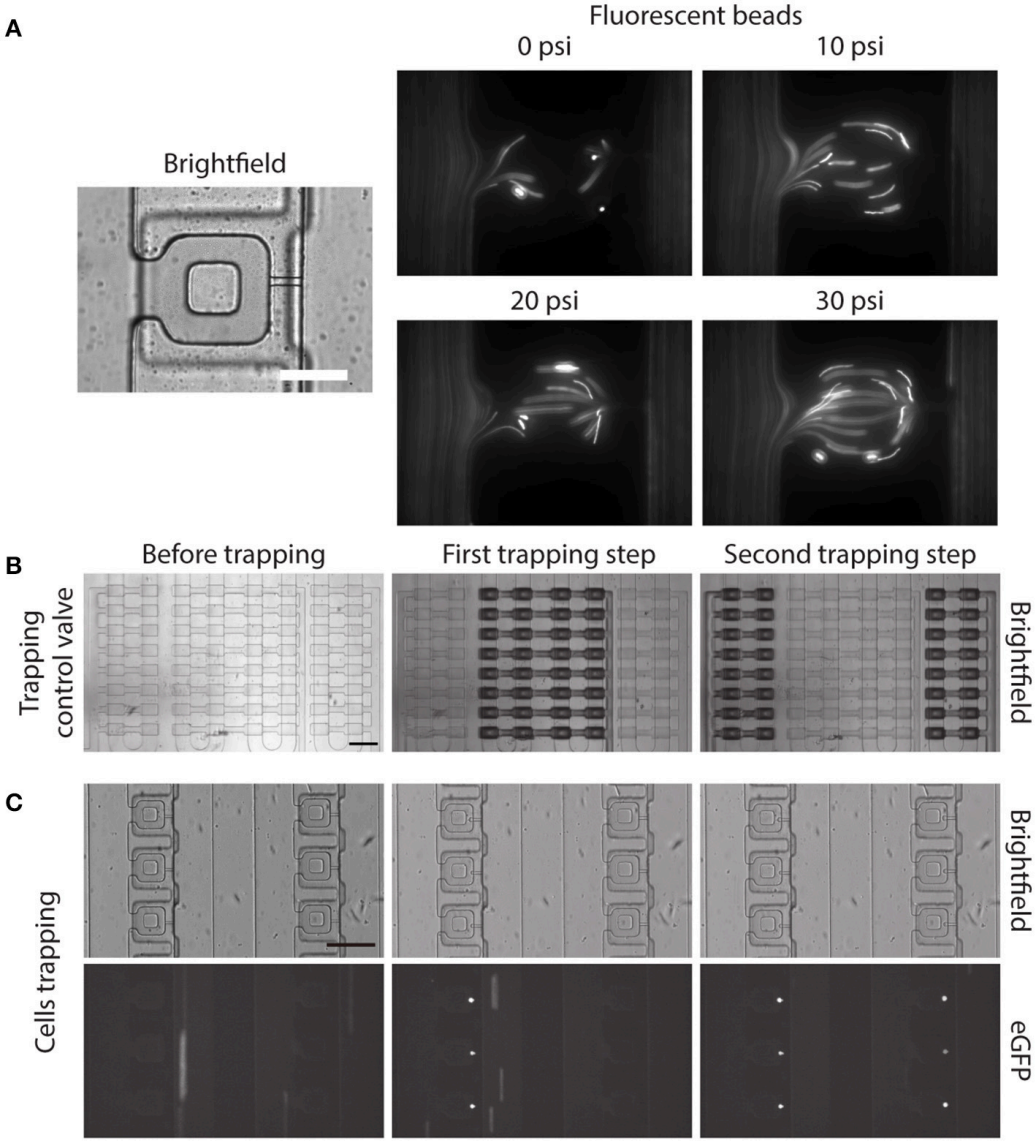
Two-step, pneumatically controlled cell trapping. **(A)** Brightfield image of the compression chamber and fluorescence images of the 1 μm Y (yellow)-G (green) fluorescent beads when the trapping control valve was pressurized at different pressures. Scale bar = 60 μm. **(B,C)** Brightfield images of the compression chamber **(B)** and fluorescence images of the trapped eGFP expressing MCF-10A cells **(C)** when the trapping control valve was changed from before trapping (**C**, left) to after first trapping step (**C**, middle) and second trapping step (**C**, right). Scale bar = 200 μm.

Before demonstrating the two-step, pneumatically controlled cell trapping, we first examined the trapping efficiency of the device when the trapping control valve was pressurized. We imaged the flow streamline inside the main microfluidic channel and the compression chamber by following the trajectories of small fluorescent beads. With increasing pressure applied in the trapping control valve, the fluid flow resistance in the main microfluidic channel increased. By the least flow resistance theory, more fluid was directed into the compression chamber and passed through the small microchannel, as shown in Figure 4A.

Next, we separately control the trapping control valves, by applying 30 psi to the trapping control valve 1 and 2 separately, to achieve the two-step pneumatically controlled cell trapping (Figure 4B). When only trapping control valve 1 was actuated, cells became trapped in the compression chamber in the first column (Figure 4C, middle). After that, trapping control valve 1 was turned to OFF and trapping control valve 2 was actuated, which facilitated cell trapping in the second column (Figure 4C, right). This control sequence demonstrated the two-step, pneumatically controlled cell trapping and a similar design strategy can be applied for a larger number of columns (i.e., increasing the number of cell trapping steps) while minimizing aspiration to the already trapped cells.

### Alignment of fibronectin island with compression block

**Figure 5:**
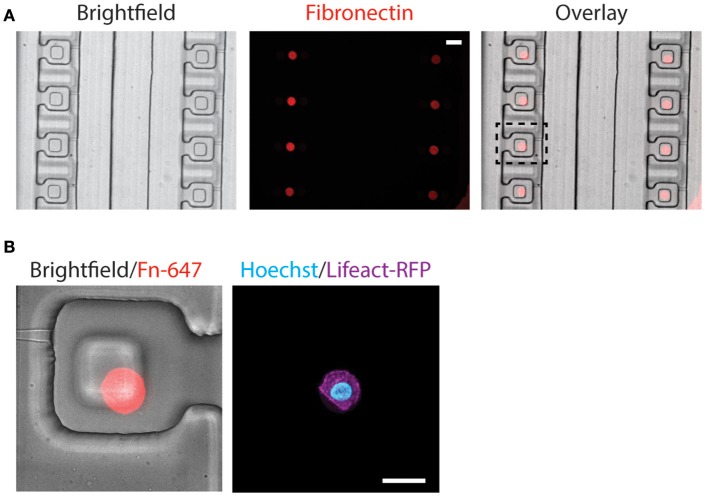
Alignment of microcontact-printed fibronectin for the attachment and compression of cells. **(A)** Brightfield and fluorescence images of the device and fibronectin, respectively. Scale bar = 50 μm. **(B)** Zoomed-in brightfield and fluorescence image of the device and fibronectin, respectively (left). Fluorescence image of the MCF-10A cell, labeling the DNA (cyan) and actin (magenta) (right). Scale bar = 20 μm.

The alignment of microcontact printed fibronectin islands with the compression block within the compression chamber is very important, as the cells need to be positioned beneath the compression block for uniform compression. Since our customized alignment platform does not support fluorescence imaging, the alignment between microcontact printed fibronectin islands and the compression chambers was achieved *via* a two-step alignment process by using a reference bottom alignment layer (detail described in the Methods section). The procedure was effective and fluorescent fibronectin colocalized well with the compression block in a fully assembled device (Figure 5A).

Following the alignment of the microcontact printed fibronectin to the compression chamber, we verified that the fabrication steps and cell trapping methods did not affect cell spreading on the fibronectin islands. MCF-10A stably expressing Lifeact-RFP was introduced to the compression device and found spread on fibronectin islands (Figure 5B). Since two different alignment steps and human eyes were involved in assembling the device, misalignment happens and the microcontact printed island does not always align in the center (Figure 5B). The PDMS block was designed to be larger than the microcontact printed island (40 μm comparing to 16 μm). It was designed to remain flat when the membrane deflects, and we further verified that in simulation and experiment (Figures 2C,D,F). Therefore, small misalignment was allowed. As long as the cell is adhered underneath the block, it will be compressed as desired. This demonstrated that the device is suitable for single-cell capture and subsequent compression.

Despite cells can spread well on microcontact printing islands, and our cell trapping efficiency is nearly 100%, we typically do not find too many cells on fibronectin islands within a fully assembled device. We attribute this low efficiency to the fact that cells that entered the trapping chamber needed to land on the fibronectin island by chance. When moving the device back to the incubator after cell trapping, cells may escape from the trapping chamber. We typically found one out of four trapped cells successfully spread on the microcontact printing islands. Even though this is not ideal, having a large number of trapping chambers helped increase the chance of having cells positioned on microcontact printed islands within the trapping chambers.

### Live cell cyclic compression

**Figure 6:**
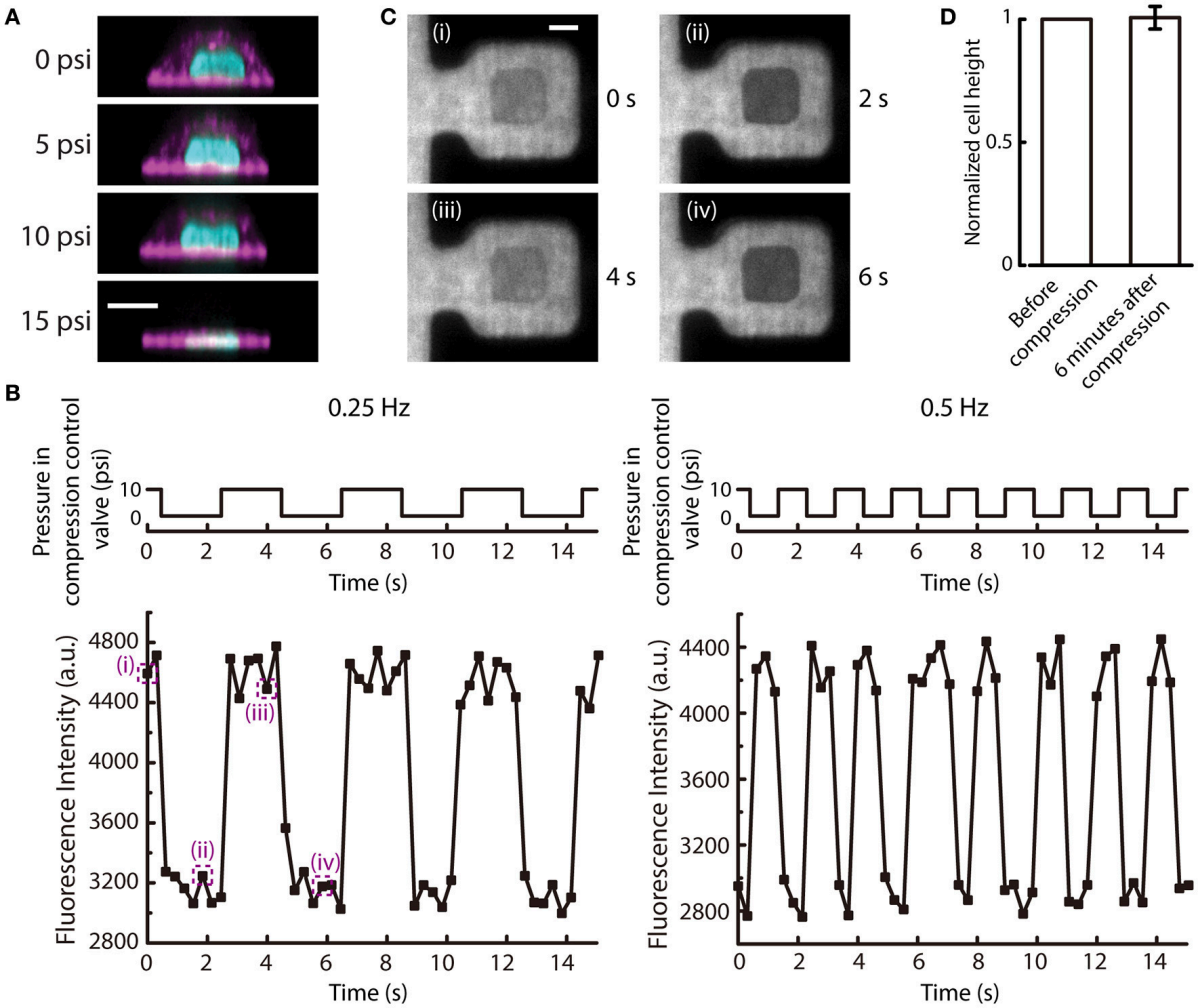
Cyclic deflection of the membrane and compression of cells. **(A)** Reconstructed side view images of a MCF-10A cell at different applied pressures to compression control valve. Cyan: DNA; Magenta: actin. Scale bar = 10 μm. **(B)** (Top) The applied cyclic pressure in the compression control valve over time at 0.25 Hz (left) and 0.5 Hz (right). (Bottom) The measured fluorescence intensity of the rhodamine succinimidyl dye at the middle of the compression chamber over time when the compression control valves were pressurized at the corresponding frequencies. **(C)** Fluorescence images of the compression chamber at different time points, as indicated in **(B)**. Scale bar = 20 μm. **(D)** The normalized cell height before and 6 min after 6-min cyclic compression alternating between 10 and 15 psi at 0.5 Hz (*n* = 6). Error bar denotes the standard error of mean. No statistical significance by *t*-test.

The new microfluidic device was designed to apply uniform compression on cells by increasing the air pressure applied to the compression control valve. This allows the cell to be compressed by the deflection of the PDMS membrane. As the pressure of the compression control valve increases, the PDMS membrane first deflected and touched the top of the cell. Then, the cell was compressed at 10 psi slightly and further compressed at 15 psi, as shown in the reconstructed side view images of the MCF-10A cell at different applied pressures (Figure 6A). This demonstrated the ability of the device to control different extent of compression on cells. The compression of cells was determined by the PDMS block pressing down on top of the cells. Therefore, due to the differences in cell height between different cells, the compressive strain that is applied to each cell will be different (may range from 0.2 to 0.8). However, all cells will have the same deformed height which is determined by the pressured applied to the compression control valve. Hence, this device will control for the same deformed height among a group of cells with different initial heights.

When the pressure in the compression control valve is alternating between high and low values, the cell can experience repetitive compression and relaxation cycle. We demonstrated that the PDMS membrane can alternate between deflected and relaxed states at different frequencies (Figure 6B). With a fluorescent dye perfused into the microfluidic device, the deflection of the membrane can be visualized. When the membrane is deflected, the fluorescence intensity in the middle of the compression chamber will reduce because the deflected PDMS block displaces the fluorescent dye. The fluorescence intensity at the middle of the compression chamber cycled between low and high intensities at a frequency of pressure application to the compression control valve (Figures 6B,C).

The mechanical behavior of living cells depends on the organization and dynamics of the cytoskeleton. The tensegrity model of living cells prescribes an interconnected network of actin filaments and microtubules that stabilizes prestress and bear compression (Wang et al., 2001). Mechanical behaviors of cells have also been compared to colloidal glass transition, in which osmotically compressed cells become stiffer and have slow intracellular relaxation (Zhou et al., 2009). In this model, the cytoskeleton is thought to have an independent and additive contribution to the stiffness changes of a cell. As a proof-of-demonstration experiment of our new compression device, we carried out cyclic compression between 10 and 15 psi on MCF-10A cells. Following 6 min of cyclic compression at 0.5 Hz, we found no statistically significant difference in cell height compared to before compression (Figure 6D), suggesting that MCF-10A cells do not experience permanent change in cell height after of cyclic compression under this condition. This result is different from the previous findings that showed cells have plastic response when a cyclic load was removed (Bonakdar et al., 2016). Since the cytoskeleton in a cell is anisotropic, with cytoskeletal filaments organized in different directions (Hu et al., 2003), cyclic loading will stretch and compress cytoskeletal fibers. When a cyclic mechanical loading is applied, tensed regions will undergo plastic deformation due to rupture of bonds, while compressed regions will not recover completely due to the inability to generate sufficient restoring forces after most of the elastic stresses have been evaded through buckling of cytoskeletal fibers (Bonakdar et al., 2016). The difference between our findings could be due to a number of reasons. First, in our device, the cells were seeded on fibronectin printed islands, which could impair the assembly of actin cytoskeleton. However, we have imaged the actin structures inside the cells when they were seeded on the fibronectin printed islands before and after being compressed. Actin stress fibers were found to form at the adhered surface (Figure S4). Second, in the Bonakdar et al. study, force was applied to a magnetic bead in both push and pull directions, rather than planar compression over a large cell area as in our case. Further, the magnetic tweezer approach was operated in a constant force mode, thus allowing the observation of increased residual deformation with increasing force cycle numbers. Our microfluidic compression device provides a distance clamp (assuming the cell does not provide a strong resistance force against the deflection membrane). When MCF-10A cells experienced cyclic planar compression, the height of the cells recovered fully. Here, we did not have high vertical resolution and temporal resolution in imaging and it was thus difficult to determine the rate of viscoelastic relaxation. We also did not have a large number of samples. This was mainly limited by the low seeding efficiency and long 3D image acquisition time of a single cell, reducing the number of cells that can be compressed and imaged at the same time. As a proof-of-concept device, one can imagine expanding the number of trapping structures to compensate for the low seeding efficiency (trapping efficiency is 100%) in the future. More detailed investigation of cytoskeleton responses during and after cyclic compression would also be an interesting future direction.

Finally, it is worth noting that there are analogous studies with cell stretching. Static cell stretching has been shown to reinforce focal adhesions at short time scale as well as delay focal adhesion disassembly at long time scale (Chen et al., 2013; Shao et al., 2013). Interestingly, the delayed response depends on the orientation of cell stretching (Chen et al., 2013). Cyclic stretching, on the other hand, elicits cell reorientation to a uniform angle that is driven by minimizing the cells' elastic energy (Livne et al., 2014). Cellular response to static or cyclic compression may also elicit different responses, and these remain to be thoroughly investigated.

## Conclusion

In this work, we developed a valve-based microfluidic device for applying compression on single adherent cells. The microfluidic device was engineered with PDMS block in the membrane to ensure optimal compression on cells and microcontact printed fibronectin in the compression chamber to control the cell spreading location. The microfluidic device was also equipped with two-step, pneumatically controlled cell trapping, to increase the number of cells that can be trapped in a device, while reducing the applied aspiration on cells during cell trapping. We have also demonstrated the application of cyclic compression on normal breast epithelial cells. The device has a unique property of compressing different cells to the same deformed height and can be easily combined with fluorescence live cell imaging. It can be used to compress different cell types with similar cell sizes used in the present work. Thus, it is possible to compare normal vs. diseased cell types to gain further insights into specific diseases. The development provides new opportunities for investigating mechanical compression in cell mechanics and mechanobiology.

## Author contributions

KH conceived the study, designed and fabricated the device, carried out experiments, analyzed data, prepared figures, and wrote the manuscript. YW performed experiments, analyzed data, and prepared figures. JW performed experiments, analyzed data, and prepared figures. AL conceived the study and wrote the manuscript.

### Conflict of interest statement

The authors declare that the research was conducted in the absence of any commercial or financial relationships that could be construed as a potential conflict of interest.

## References

[B1] Bausch A. R. Hellerer U. Essler M. Aepfelbacher M. Sackmann E. (2001). Rapid stiffening of integrin receptor-actin linkages in endothelial cells stimulated with thrombin: a magnetic bead microrheology study. Biophys. J. 80, 2649–2657. 10.1016/S0006-3495(01)76234-0 11371441 PMC1301452

[B2] Bonakdar N. Gerum R. Kuhn M. Spörrer M. Lippert A. Schneider W.. (2016). Mechanical plasticity of cells. Nat. Mater. 15, 1090–1094. 10.1038/nmat4689 27376682

[B3] Chen Y. Pasapera A. M. Koretsky A. P. Waterman C. M. (2013). Orientation-specific responses to sustained uniaxial stretching in focal adhesion growth and turnover. Proc. Natl. Acad. Sci. U.S.A. 110, E2352– E2361. 10.1073/pnas.1221637110 23754369 PMC3696741

[B4] Denais C. M. Gilbert R. M. Isermann P. McGregor A. L. te Lindert M. Weigelin B.. (2016). Nuclear envelope rupture and repair during cancer cell migration. Science 352, 353–358. 10.1126/science.aad7297 27013428 PMC4833568

[B5] Fabry B. Maksym G. N. Butler J. P. Glogauer M. Navajas D. Fredberg J. J. (2001). Scaling the microrheology of living cells. Phys. Rev. Lett. 87:148102. 10.1103/PhysRevLett.87.148102 11580676

[B6] Grodzinsky A. J. Levenston M. E. Jin M. Frank E. H. (2000). Cartilage tissue remodeling in response to mechanical forces. Annu. Rev. Biomed. Eng. 2, 691–713. 10.1146/annurev.bioeng.2.1.691 11701528

[B7] Gupta V. Grande-Allen K. J. (2006). Effects of static and cyclic loading in regulating extracellular matrix synthesis by cardiovascular cells. Cardiovasc. Res. 72, 375–383. 10.1016/j.cardiores.2006.08.017 17010955

[B8] Heureaux J. Luker K. E. Haley H. Pirone M. Lee L. M. Liu A. P. (2018). The effect of mechanosensitive channel MscL expression in cancer cells on 3D confined migration. APL Bioeng. 2:032001 10.1063/1.5019770 PMC6324216 31069318

[B9] Ho K. K. Lee L. M. Liu A. P. (2016). Mechanically activated artificial cell by using microfluidics. Sci. Rep. 6:32912. 10.1038/srep32912 27610921 PMC5017192

[B10] Hosmane S. Fournier A. Wright R. Rajbhandari L. Siddique R. Yang I.. (2011). Valve-based microfluidic compression platform: single axon injury and regrowth. Lab Chip 11, 3888–3895. 10.1039/c1lc20549h 21975691

[B11] Hu S. Chen J. Fabry B. Numaguchi Y. Gouldstone A. Ingber D.. (2003). Intracellular stress tomography reveals stress focusing and structural anisotropy in cytoskeleton of living cells. Am. J. Physiol. Cell Physiol. 285, C1082–C1090. 10.1152/ajpcell.00159.2003 12839836

[B12] Jain R. K. Martin J. D. Stylianopoulos T. (2014). The Role of mechanical forces in tumor growth and therapy. Annu. Rev. Biomed. Eng. 16, 321–346. 10.1146/annurev-bioeng-071813-105259 25014786 PMC4109025

[B13] Kim Y. C. Kang J. H. Park J-S. Yoon E-S, Park, K-J. (2007). Microfluidic biomechanical device for compressive cell stimulation and lysis. Sens. Actuators B Chem. 128, 108–116. 10.1016/j.snb.2007.05.050

[B14] Lee L. M. Lee J. W. Chase D. Gebrezgiabhier D. Liu A. P. (2016). Development of an advanced microfluidic micropipette aspiration device for single cell mechanics studies. Biomicrofluidics 10:054105. 10.1063/1.4962968 27703591 PMC5035296

[B15] Li Z. Li X. McCracken B. Shao Y. Ward K. Fu J. (2016). A miniaturized hemoretractometer for blood clot retraction testing. Small 12, 3926–3934. 10.1002/smll.201600274 27248117 PMC4980575

[B16] Liu A. P. (2016). Biophysical tools for cellular and subcellular mechanical actuation of cell signaling. Biophys. J. 111, 1112–1118. 10.1016/j.bpj.2016.02.043 27456131 PMC5034300

[B17] Liu A. P. Chaudhuri O. Parekh S. H. (2017). New advances in probing cell-extracellular matrix interactions. Integr. Biol. 9, 383–405. 10.1039/C6IB00251J 28352896 PMC5708530

[B18] Livne A. Bouchbinder E. Geiger B. (2014). Cell reorientation under cyclic stretching. Nat. Commun. 5:3938. 10.1038/ncomms4938 24875391 PMC4066201

[B19] Lulevich V. Yang H. Y. Rivkah Isseroff R. Liu G. Y. (2010). Single cell mechanics of keratinocyte cells. Ultramicroscopy 110, 1435–1442. 10.1016/j.ultramic.2010.07.009 20728993

[B20] Lulevich V. Zink H-T. Chen H. Y. Liu T. Liu G. Y. (2006). Cell mechanics using atomic force microscopy-based single-cell compression. Langmuir 22, 8151–8155. 10.1021/la060561p 16952255

[B21] Mammoto T. Ingber D. E. (2010). Mechanical control of tissue and organ development. Development 137, 1407–1420. 10.1242/dev.024166 20388652 PMC2853843

[B22] Polacheck W. J. Li R. Uzel S. G. Kamm R. D. (2013). Microfluidic platforms for mechanobiology. Lab Chip 13, 2252–2267. 10.1039/c3lc41393d 23649165 PMC3714214

[B23] Raab M. Gentili M. de Belly H. Thiam H. R. Vargas P. Jimenez A. J.. (2016). ESCRT III repairs nuclear envelope ruptures during cell migration to limit DNA damage and cell death. Science 352, 359–362. 10.1126/science.aad7611 27013426

[B24] Reymond N. d'Agua B. B. Ridley A. J. (2013). Crossing the endothelial barrier during metastasis. Nat. Rev. Cancer 13, 858–870. 10.1038/nrc3628 24263189

[B25] Ricca B. L. Venugopalan G. Furuta S. Tanner K. Orellana W. A. Reber C. D.. (2018). Transient external force induces phenotypic reversion of malignant epithelial structures via nitric oxide signaling. Elife 7:e26161. 10.7554/eLife.26161 29560858 PMC5862525

[B26] Rosenbluth M. J. Lam W. A. Fletcher D. A. (2006). Force microscopy of nonadherent cells: a comparison of leukemia cell deformability. Biophys. J. 90, 2994–3003. 10.1529/biophysj.105.067496 16443660 PMC1414579

[B27] Shao Y. Tan X. Novitski R. Muqaddam M. List P. Williamson L.. (2013). Uniaxial cell stretching device for live-cell imaging of mechanosensitive cellular functions. Rev. Sci. Instrum. 84:114304. 10.1063/1.4832977 24289415 PMC3862604

[B28] Shyy J. Y. Chien S. (2002). Role of integrins in endothelial mechanosensing of shear stress. Circ. Res. 91, 769–775. 10.1161/01.RES.0000038487.19924.18 12411390

[B29] Si F. Li B. Margolin W. Sun S. X. (2015). Bacterial growth and form under mechanical compression. Sci. Rep. 5:11367. 10.1038/srep11367 26086542 PMC4471898

[B30] Van Vliet K. J. Bao G. Suresh S. (2003). The biomechanics toolbox: experimental approaches for living cells and biomolecules. Acta Mater. 51, 5881–5905. 10.1016/j.actamat.2003.09.001

[B31] Wang N. Naruse K. Stamenović D. Fredberg J. J. Mijailovich S. M. Tolić-Nørrelykke I. M.. (2001). Mechanical behavior in living cells consistent with the tensegrity model. Proc. Natl. Acad. Sci. U.S.A. 98, 7765–7770. 10.1073/pnas.141199598 11438729 PMC35416

[B32] Weafer P. P. Ronan W. Jarvis S. P. McGarry J. P. (2013). Experimental and computational investigation of the role of stress fiber contractility in the resistance of osteoblasts to compression. Bull. Math. Biol. 75, 1284–1303. 10.1007/s11538-013-9812-y 23354930

[B33] Xia Y. Whitesides G. M. (1998). Soft lithography. Annu. Rev. Mater. Sci. 28, 153–184. 10.1146/annurev.matsci.28.1.153

[B34] Zhou E. H. Trepat X. Park C. Y. Lenormand G. Oliver M. N. Mijailovich S. M.. (2009). Universal behavior of the osmotically compressed cell and its analogy to the colloidal glass transition. Proc. Natl. Acad. Sci. U.S.A. 106, 10632–10637. 10.1073/pnas.0901462106 19520830 PMC2695406

