# Supplementary material for: Advanced Microfluidic Device Designed for Cyclic Compression of Single Adherent Cells
Source: Front Bioeng Biotechnol. 2018 Oct 16;6:148. doi: 10.3389/fbioe.2018.00148 (PMC6198036; doi:10.3389/fbioe.2018.00148)
Supplement: Supplementary file 1 [file Data_Sheet_1.pdf]

Supplemental Materials

Advanced microfluidic device designed for cyclic compression of single adherent cells

Kenneth K.Y. Ho[1,2], Ying Lin Wang[1,3], Jing Wu[1,4], Allen P. Liu[1,5,6,7]

[1] Department of Mechanical Engineering, University of Michigan, Ann Arbor, Michigan, United States of America, [4] Department of Mechanical Engineering, University of Hong Kong, Hong Kong, [5] Department of Biomedical Engineering, University of Michigan, Ann Arbor, Michigan, United States of America, [6] Cellular and Molecular Biology Program, University of Michigan, Ann Arbor, Michigan, United States of America, [7] Biophysics Program, University of Michigan, Ann Arbor, Michigan, United States of America.

[2] present address: Biocrede, Plymouth, MI, U.S.A.

[3] present address: Trust Bio-sonics, Hsinchu, Taiwan

[*] Corresponding author: Allen Liu, Ph.D., University of Michigan, Ann Arbor, 2350 Hayward Street, MI 48105. Tel: 734-764-7719, Email: allenliu@umich.edu

Supplemental Materials

Device fabrication – SU-8 patterning

Three photomasks were produced by high resolution inkjet printing on transparency film (CAD/Art Services). Four silicon molds were SU-8 patterned by standard photolithography using the three photomasks. The first silicon mold is composed of one layer of SU-8 pattern for PDMS casting of the control layer. The SU-8 pattern of the control layer defined two sets of integrated microfluidic control valves for closing of the main microfluidic channel (trapping control valve) and deflection of the flow layer in the compression chambers (compression control valve) respectively. The second silicon mold is composed of three layers of SU-8 patterns to be used for PDMS spin-coating of the flow layer. The first SU-8 patterning layer defined the side microfluidic pipette channel for trapping cells. The second SU-8 patterning layer defined the compression block for flat planar compression of cells. The third SU-8 patterning layer defined the main microfluidic channel, the trapping chambers, the inlets and the outlet. The third silicon mold is composed of one layer of SU-8 pattern for PDMS casting of the bottom alignment layer. The SU-8 pattern of the bottom alignment layer has the same design as the third SU-8 patterning layer of the second silicon mold, so that the bottom alignment layer can be used for aligning the fibronectin and the PDMS control/flow substrate. The fourth silicon mold is composed of one layer of SU-8 pattern for PDMS casting of the microcontact printing layer. The microcontact printing layer defines the shape and size for printing fibronectin directly within the compression chambers.

The SU-8 patterning procedures of the four silicon molds followed the standard protocol developed by Microchem, Inc. for SU-8 2000. First, four silicon wafers were dehydrated on a hot plate at 200°C for 15 min to promote photoresist adhesion. For SU-8 patterning the silicon mold for the control layer, SU-8 2025 was spin-coated at 4000 rpm onto the wafer for 30 s, which gave a thickness of 20 µm. The photoresist was then exposed to UV light for 12 s under a contact aligner (Karl Suss, MJB45). For the first SU-8 patterning of the flow layer, SU-8 2005 was spin-coated at 2700 rpm onto the silicon wafer for 30 s, which gave a thickness of 5.4 µm. The photoresist was then exposed to UV light for 9 s with the first flow channel photomask, which defined the side pipette arrays. After development of first SU-8 layer, the silicon wafer was hard-baked at 200°C for 20-30 minutes to further cross-link the developed SU-8 patterns before the application of the second SU-8 layer. Subsequently, for the second-layer SU-8 patterning of the flow layer, SU-8 2025 was spin-coated at 3500 rpm onto the silicon wafer for 30 s, which gave a combined thickness of 24 µm. The photoresist was then aligned with the second flow channel photomask, which defined the compression block, and exposed to UV light

for 11.5 s. After development of SU-8, both silicon molds were hard-baked at 200°C for 20-30 minutes to further cross-link the developed SU-8 patterns before the application of the third SU-8 layer. Subsequently, for the third-layer SU-8 patterning of the flow layer, SU-8 2025 was spin-coated at 2100 rpm onto the silicon wafer for 30 s, which gave a combined thickness of 38 μm. The photoresist was then aligned with the third flow channel photomask, which defined the main microfluidic channel, and exposed to UV light for 12.5 s. For SU-8 patterning the silicon mold for the bottom alignment layer, SU-8 2010 was spin-coated at 2000 rpm onto the wafer for 30 s, which gave a thickness of 13 μm. The photoresist was then exposed to UV light for 9 s under a contact aligner. For SU-8 patterning the silicon mold for the microcontact printing layer, SU-8 2025 was spin-coated at 4000 rpm onto the wafer for 30 s, which gave a thickness of 20 μm. The photoresist was then exposed to UV light for 12 s under a contact aligner. After development of SU-8, all four silicon molds were hard-baked at 200°C for 20-30 minutes again to cure any surface cracks. The thicknesses of both the control layer and flow channel SU-8 pattern were measured using a stylus profilometer (Dektak 6M).

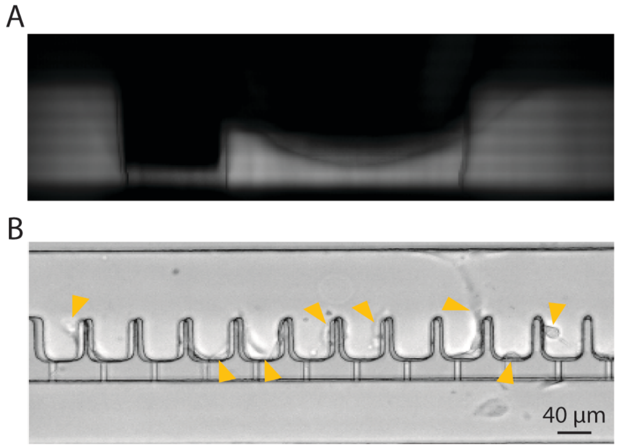

Figure S1: Potential difficulties with previous microfluidic compression device for single cell compression. (A) Side view image of rhodamine succinimidyl dye perfused in the microfluidic channel reconstructed in ImageJ. **(B)** Brightfield image showed random cell spreading inside the microfluidic device, as pointed out by the arrowhead.

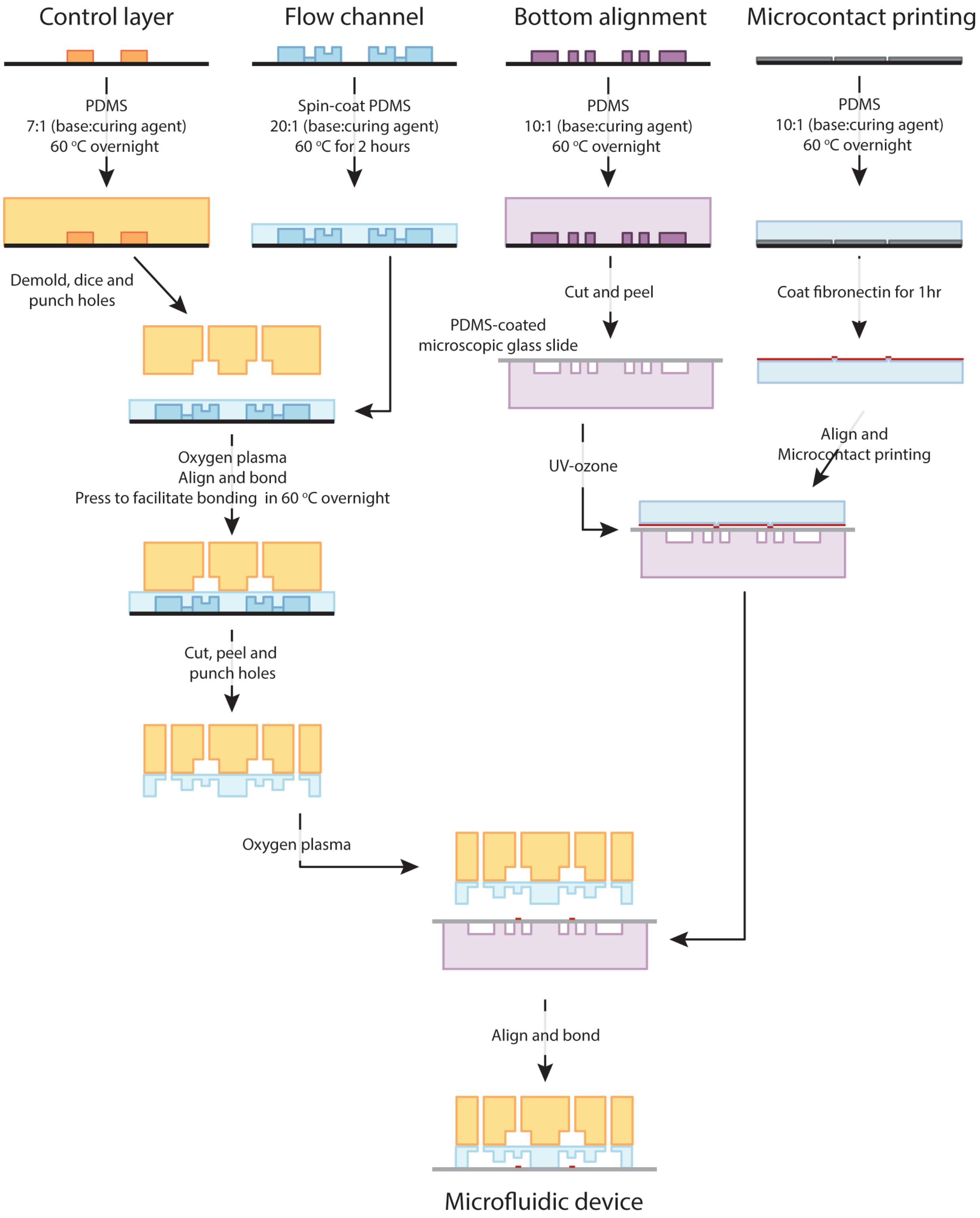

Figure S2: Fabrication process of microfluidic device. Four silicon molds for control layer, flow channel, bottom alignment layer and microcontact printing layer were casted with PDMS, demolded, cut, aligned, bonded, surface treated at different stages.

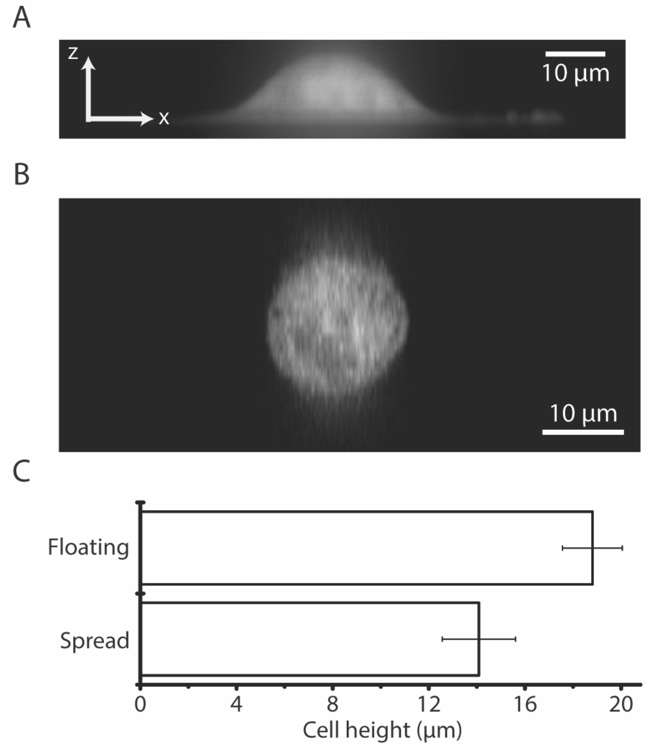

Figure S3: Side view and height of MCF-10A cell in suspension and attached on a surface. (A) Reconstructed side view fluorescence image of an attached MCF-10A cell. **(B)** Reconstructed side view fluorescence image of a MCF-10A cell in suspension. **(C)** The average height of MCF-10A cells when floating (n = 13) or spread on a surface (n = 13). Error bar represents S.E.M..

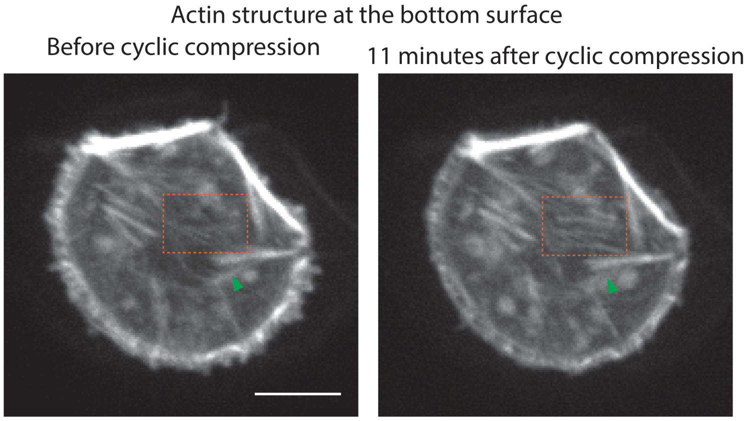

Figure S4: Actin structure at the bottom surface of a MCF-10A cell before and after cyclic compression. Fluorescence image of an attached MCF-10A cell labeling actin filaments by Lifeact. Dotted boxed region showed formation of new stress fibers. Green arrowhead points to thickening of a pre-existing actin structure. Scale bar = 10 μm.
